# Supplementary material for: Single-Molecule X-ray Scattering Used to Visualize the Conformation Distribution of Biological Molecules via Single-Object Scattering Sampling
Source: Int J Mol Sci. 2023 Dec 5;24(24):17135. doi: 10.3390/ijms242417135 (PMC10743147; doi:10.3390/ijms242417135)
Supplement: Supplementary file 1 [file ijms-24-17135-s001.zip › ijms-2722754-supplementary.pdf]

Supplementary Information for

Single-Molecule X-ray Scattering Used to Visualize the Conformation Distribution of Biological Molecules via Single-Object Scattering Sampling

Seonggon Lee[1,2,†], Hosung Ki[1,2,†], Sang Jin Lee[1,2] and Hyotcherl Ihee[1,2,*]

[1] Department of Chemistry and KI for the BioCentury, Korea Advanced Institute of Science and Technology (KAIST), Daejeon 305-701, Republic of Korea
[2] Center for Advanced Reaction Dynamics (CARD), Institute for Basic Science (IBS), Daejeon, 34141, Republic of Korea
* Correspondence: hyotcherl.ihee@kaist.ac.kr
† These authors contributed equally to this work.

Supporting Methods

1. X-ray scattering simulation of AuNP-labeled ssRNA

1-1. AuNP labeling

In our simulations, we positioned two AuNPs with a predefined radius, *R*, at a randomly selected pair of sites in a target biomolecule. For RNAs, we identified potential substitution sites for the AuNPs as the two free oxygen atoms, "O1P" and "O2P" (also referred to as "OP1" and "OP2" in some PDB files), located within the phosphate groups linking nucleotides. As an example, the RNA structure with PDB ID 1KXK, which is composed of 69 phosphorus atoms, offers 138 × 136 / 2 = 9,384 potential oxygen atoms pairs (O, O) for AuNP substitution. In proteins, the oxygen atoms within the peptide bonds (–CO–NH–) were selected as the potential sites for AuNP substitution. For our substitution methodology, the center of mass of each AuNP was directly placed at the selected oxygen atom, bypassing the use of additional linkers between the labeling site and the AuNP. We note that this approach does not reflect the typical realistic scenario of AuNP labeling on biomolecules where AuNPs are commonly attached to specific sites on biomolecules using lengthy linker molecules. Nevertheless, considering our objective in this work is to showcase the potential of Bio-SOSS in retrieving the distance distribution of biomolecules labeled with AuNPs, we opted for a simplified model. Given the relatively minor contribution of the biomolecules and any linkers to the overall scattering pattern, this simplified approach was deemed both efficient and sufficient for the intended purpose of demonstrating Bio-SOSS's capabilities. An exclusion criterion was also implemented to avoid scenarios where the distance between two AuNPs was too close, specifically when the distance between their centers, r_{AuNP}, was less than 2.4 times the radius *R*.

In **Figure 3** and **Figures S4 and S5**, we present a quantitative analysis of the accuracy of Bio-SOSS using molecular dynamics (MD) snapshots of the RNA molecule with PDB ID: 1KXK. For this analysis, we randomly selected 18 pairs from the total available pairs, and compared the distribution of distances retrieved from Bio-SOSS with those obtained from 100 MD snapshots. In **Figure 4**, we statistically considered a total of 1,800 distances (18 pairs × 100 snapshots) to determine statistical parameters, notably the standard deviation of the distance differences, denoted as $\sigma(\Delta r)$. Further, in **Figures S6 and S18–S21**, we assessed the efficacy of Bio-SOSS in ten different biomolecules, as listed in **Figure S1**. This evaluation involved the generation and the analysis of X-ray scattering images from 30 distinct orientations for each biomolecule, using their fixed structures as reported in the corresponding PDB entries.

1-2. Generation of single-object scattering images

When a molecule scatters X-ray pulses, the scattered photons generate unique X-ray scattering patterns that are dependent upon the structure between atomic scatterers. Therefore, these X-ray scattering images can hypothetically be simulated when the structure of a molecule exposed to an X-ray irradiation is known. In our case, hypothetical X-ray scattering images, denoted as S_{exp}, were calculated for the instantaneous structures of the single molecular AuNP-labeled RNA. On the other hand, X-ray scattering solely from the two AuNPs distanced by an iterable 3D distance was calculated and labeled as S_{theo}. In the calculation, we assumed that the scattered photons were collected on a two-dimensional area detector using the following equation.

$$\mathbf{S}(\boldsymbol{u}, \Omega) = 0.5\,(1 + \cos^2 2\theta) \cdot \Omega \cdot r_e \cdot \int \mathbf{I}(t) \cdot \Sigma_j\, f_j(t) \cdot |\exp^{i\Delta k(\boldsymbol{u})\cdot x_j(t)}|^2\, dt \tag{S1}$$

Here, u is the position vector of a detector pixel, Ω is the solid angle of the detector pixel, θ is a half of the scattering angle corresponding to the pixel, r_e is the classical radius of an electron, $\mathbf{I}(t)$ is the instant photon flux at time t, $f_j(t)$ is the atomic form factor of the jth atom at time t, $\Delta k(\boldsymbol{u})$ is the change in the scattering vector, and $x_j(t)$ is the position vector of the jth atom at time t. This equation calculates the number of elastically scattered photons. Incoherent scattering is ignored in this calculation. To calculate the scattering intensity detected at each pixel, we considered the intensity only at the center of the pixel and assumed that the intensity is equal at all different positions within one pixel. The form factors of the nanoparticles were approximated as follows.

$$f_{AuNP}(q, R) = 3\, f_{Au}(q) \cdot [\, \sin(qR) - qR \cdot \cos(qR)\,] \cdot q^{-3} \tag{S2}$$

Here, q is the magnitude of the momentum transfer vector between the incident and elastically scattered X-ray photon, R is the radius of the nanoparticle, f_{AuNP} is the X-ray scattering form factor of the nanoparticle of radius R, and f_{Au} is the X-ray scattering form factor of a gold (Au) atom.

1-3. Optimization of 3D distances

We implemented the least-squares minimization method to extract the 3D distances between the two AuNPs. Since the theoretical X-ray scattering data only reflects the scatterings from the two AuNPs, the vector displacements from one AuNP to the other solely contribute to $S_{theo}(q_x, q_y)$. Hence, we placed one AuNP at the origin of a hypothetical lab frame and parameterized the 3D coordinates of the other AuNP with the following three variables: x_{fit}, y_{fit}, z_{fit}. Since the 3D coordinates were defined in a relative manner, we also imposed the constraint of positivity to the three parameters.

Afterward, we optimized the set of 3D distances (x_{fit}, y_{fit}, z_{fit}) that minimized the reduced chi-square, χ^2_{red}, between the experimental and theoretical X-ray scattering intensity over the entire detector of $n_x \times n_y$ pixels. The standard deviation at each pixel was determined by the sum of the static noise and the Poisson random noise. The static noise was modeled with a uniform distribution function centered at b, the scaling constant for the uniform distribution. The Poisson noise had identical value, $aS_{exp}(i,j)$, for both the expectation value and variance. Thus, the minimization function took the following formula.

$$\chi^2_{red} = \Sigma_i^{n_x} \Sigma_j^{n_y} \left(\frac{\Delta S(i,j)_{exp} - \Delta S(i,j)_{theo}}{a\Delta S(i,j)_{exp} + b} \right)^2 \tag{S3}$$

Here, n_x and i are the total number and index of detector pixels along the x axis, n_y and j are those along the y axis, a is the scaling constant for the Poisson distribution (taken to 1), and b is taken to be 0.1. We used a lab-made *MATLAB* code for the minimization.

2. Statistical assessment of the goodness-of-fit

2-1. Gaussian fit analysis

To quantitatively assess how the experimental parameters affect the accuracy of retrieved distance

distributions, we parameterized the normalized probability distribution along Δx, Δy, and Δz with a Gaussian distribution function.

$$p(\sigma_x, \mu_x, A_x; \Delta x) = \frac{A_x}{\sqrt{2\pi}\sigma_x} \exp\left(-\frac{(\Delta x - \mu_x)^2}{2\sigma_x^2}\right) \quad \text{(S4)}$$

$$p(\sigma_y, \mu_y, A_y; \Delta y) = \frac{A_y}{\sqrt{2\pi}\sigma_y} \exp\left(-\frac{(\Delta y - \mu_y)^2}{2\sigma_y^2}\right) \quad \text{(S5)}$$

$$p(\sigma_z, \mu_z, A_z; \Delta z) = \frac{A_z}{\sqrt{2\pi}\sigma_z} \exp\left(-\frac{(\Delta z - \mu_z)^2}{2\sigma_z^2}\right) \quad \text{(S6)}$$

In this equation, Δx, Δy, and Δz represent the difference between fitted and actual distances along each axis, $A_{x,y,z}$ stands for the amplitude of the Gaussian function, and μ and σ denote the average and standard deviation for the distribution of Δr. A total of n_{bins} = 200 pairs of (Δx, N_x), (Δy, N_y), and (Δz, N_z) were used to optimize the three parameters, $\mu_{x,y,z}$, $\sigma_{x,y,z}$, and $A_{x,y,z}$. Ideally, the average value $\mu_{x,y,z}$ of the difference distances should be zero if the Bio-SOSS mechanism works correctly. Therefore, the effect of the experimental parameters was reflected in the standard deviation $\sigma_{x,y,z}$ designating the precision of the distribution. In this context, we compared $\sigma_{x,y,z}$, instead of $\mu_{x,y,z}$, of Δx, Δy, Δz, and $\Delta r = ((\Delta x)^2 + (\Delta y)^2 + (\Delta z)^2)^{1/2}$ as criteria to quantitatively evaluate the accuracy at a predefined set of experimental conditions. We note that, in the Gaussian fit analysis, we omitted bins with probability counts lower than 0.5% for clarity.

3. X-ray scattering images from RNA, a pair of AuNPs, and RNA labeled with two AuNPs

We employed a step-by-step algorithm to generate the X-ray scattering images as detailed in the "Methods" section and SI. The simulated X-ray scattering images of the RNA (S_{RNA}), a pair of AuNPs (S_{AuNP}), and RNA labeled with a pair AuNPs ($S_{AuNP-RNA}$) were directly computed from each structure. The X-ray scattering image representing what can actually be measured in the experiment (S_{exp}) was generated by adding the Poisson noise and uniform noise to $S_{AuNP-RNA}$. The theoretical X-ray scattering image (S_{theo}) used to fit the experimental one was computed only from the two AuNPs, but the 3D distances between the two were iteratively optimized to best mimic S_{exp}. In **Figure S2**, four sample images for S_{RNA}, S_{AuNP}, $S_{AuNP-RNA}$, S_{exp}, and S_{theo} are presented to visually depict the logical development.

4. Visualization of the retrieved distance distributions.

In **Figures S4** and **S5**, the remaining 12 pairs for the distance distribution between nucleoside pairs are visualized. The information contained in these figures are analogous to **Figure 3**.

5. Dependence of the accuracy of Bio-SOSS on experimental parameters

In this section, we present the results of least-squares minimization with a Gaussian distribution

function (see section 2-1 of SI) at varying experimental conditions. The distribution of difference distances (Δx, Δy, and Δz) were sorted into a histogram of predefined bin sizes for Δx, Δy, and Δz, respectively. The number counts at each bin were fitted by a Gaussian distribution function with the mean value μ and standard deviation σ. Except for **Figure S13**, the Gaussian fit analyses were repeated with (i) a larger bin of 0.02 Å ranging from −2 Å to 2 Å for Δx and Δy, and from −5 Å to 5 Å for Δz, and (ii) a smaller bin of 0.01 Å ranging from −0.02 Å to 0.02 Å for Δx and Δy, and −1.2 Å to 1.2 Å for Δz. The larger bin was intended to show the general accordance between real distances from the MD snapshot and fitted ones from the Bio-SOSS. The shorter bin was intended to extract the precise μ and σ values at each case. The fit results from these histogram analyses were summarized in **Figure 6**.

Figures S7 and **S8** depict the Gaussian fit diagrams with varying AuNP sizes. **Figures S9** and **S10** show the Gaussian fit diagrams at varying X-ray photon fluxes. **Figures S11** and **S12** depict the Gaussian fit diagrams at varying X-ray wavelengths (or energies). **Figure S13** shows the Gaussian fit diagrams at varying X-ray focal sizes. **Figures S14** and **S15** depict the Gaussian fit diagrams at varying number of detector pixels. **Figures S16** and **S17** present the Gaussian fit diagrams at varying sample-to-detector distances.

6. Bio-SOSS on various biomolecules

The Bio-SOSS accuracy was quantitatively assessed with ten distinct biomolecules (**Figure S1**) comprising four RNAs, five proteins, and an RNA-protein complex. To specifically investigate how the size of the target biomolecule, rather than its conformational diversity, influences the accuracy of Bio-SOSS, we generated multiple scattering images from only a single static structure per molecule. The two variations introduced were: (1) the position of the labeling site and (2) the angular orientation of the molecule relative to the direction of X-ray propagation (details in the "Materials and Methods" section). Our results demonstrate a notable agreement between the actual distances (r_{AuNP}) and the distance retrieved by Bio-SOSS (r_{fit}) as depicted in **Figures S18–S21**. This level of agreement is comparable to that observed for the MD snapshots of the target RNA (PDB ID: 1KXK). Employing the same experimental parameters as in **Figure 4**, the standard deviation of the distance differences, $\sigma(\Delta r)$, for these ten biomolecules ranged from 0.097 Å to 0.33 Å. In addition, the $\sigma(\Delta r)$ value shows a positive correlation with the size of the biomolecules, as characterized by either the molecular weight or the number of residues (**Figure S6**). This trend can be attributed to the fact that a higher molecular weight typically results in a more pronounced X-ray scattering signal from the biological molecule, consequently reducing the relative contribution or scattering contrast provided by the two AuNPs. It is noteworthy that, while both RNAs and proteins exhibit the general trend of $\sigma(\Delta r)$ increasing with size, the degree to which $\sigma(\Delta r)$ increases with size shows distinct characteristics in RNAs and proteins. Specifically, we observed a linear increase in $\sigma(\Delta r)$ with the molecular weight of proteins. In contrast, for RNAs, the increase, or "worsening", of $\sigma(\Delta r)$ demonstrated a saturating trend with an increase in the molecular weight. We attribute this deviation to the distinct geometric dimensions of RNAs and proteins. Nucleic acids, including RNAs, exhibit directionality from the 5' end to the 3' end, resulting in a lengthy structure in one specific direction compared to the other two dimensions. On the other hand, proteins typically exhibit intricate three-dimensional structures, as opposed to elongation along a single direction, stemming from tertiary or quaternary interactions. Consequently, as the size of the biomolecule increases, the effective "thickness" encountered by incident X-rays during scattering interactions becomes notably greater in proteins compared to RNAs. In other words, the projection density along the propagation direction of the incident X-ray increases more rapidly in proteins as their size grows. In contrast, in larger RNAs, this "thickness" tends to reach a

saturation point in longer, larger RNAs. Accordingly, there is a saturation of size-dependent “worsening” effects in RNAs, a phenomenon not observed in proteins.

In this section, Supplementary Figures

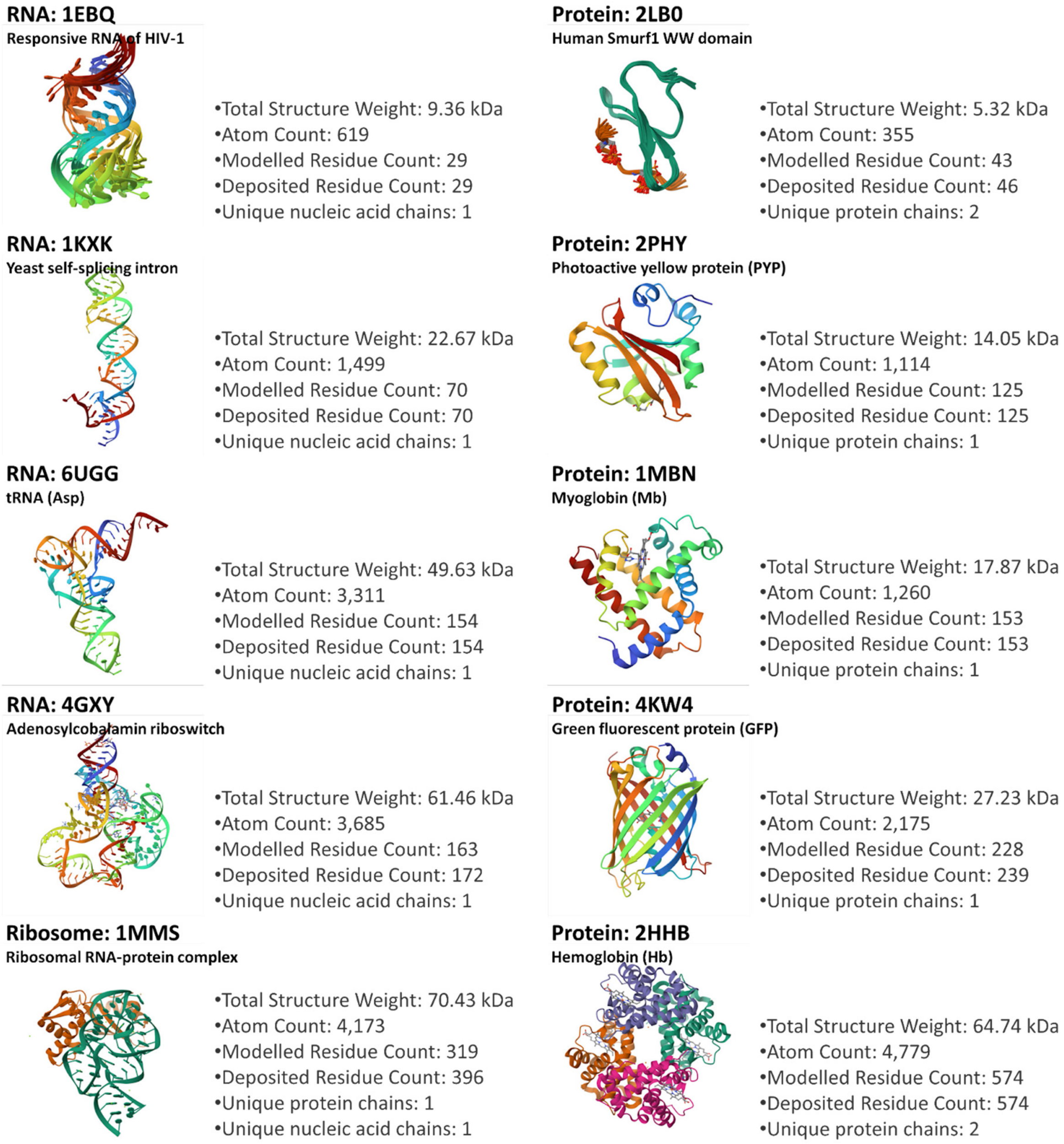

Figure S1. Information of biomolecules used for simulating Bio-SOSS images. In this work, the Bio-SOSS method was tested on four RNAs, five proteins, and one RNA-protein complex. For RNAs, (1) the rRNA of HIV-1 virus (PDB ID: 1EBQ, 9.36 kDa) [47], (2) the yeast ai5g group II intron (PDB ID: 1KXK, 22.67 kDa) [48], (3) the tRNA for the aspartylation mechanism (PDB ID: 6UGG, 49.63 kDa) [49], and (4) the adenosylcobalamin riboswitch (PDB ID: 4GXY, 61.46 kDa) [50] were used. In addition, a ribosomal RNA-protein complex (PDB ID: 1MMS, 70.43 kDa) [51] was used as an example for RNA-protein complexes. For proteins, (1) the first WW domain of human Smurf1 (PDB ID: 2LB0, 5.32 kDa) [52], (2) photoactive yellow protein (PDB ID: 2PHY, 14.05 kDa) [53], (3) myoglobin (PDB ID: 1MBN, 17.87 kDa) [54], (4) a single unit of green fluorescent protein (PDB ID: 4KW4, 27.23 kDa) [55], and (5) hemoglobin (PDB ID: 2HHB, 64.74 kDa) [56] were used. The visual depiction and chemical information related to each molecule were replicated from the RCSB website.

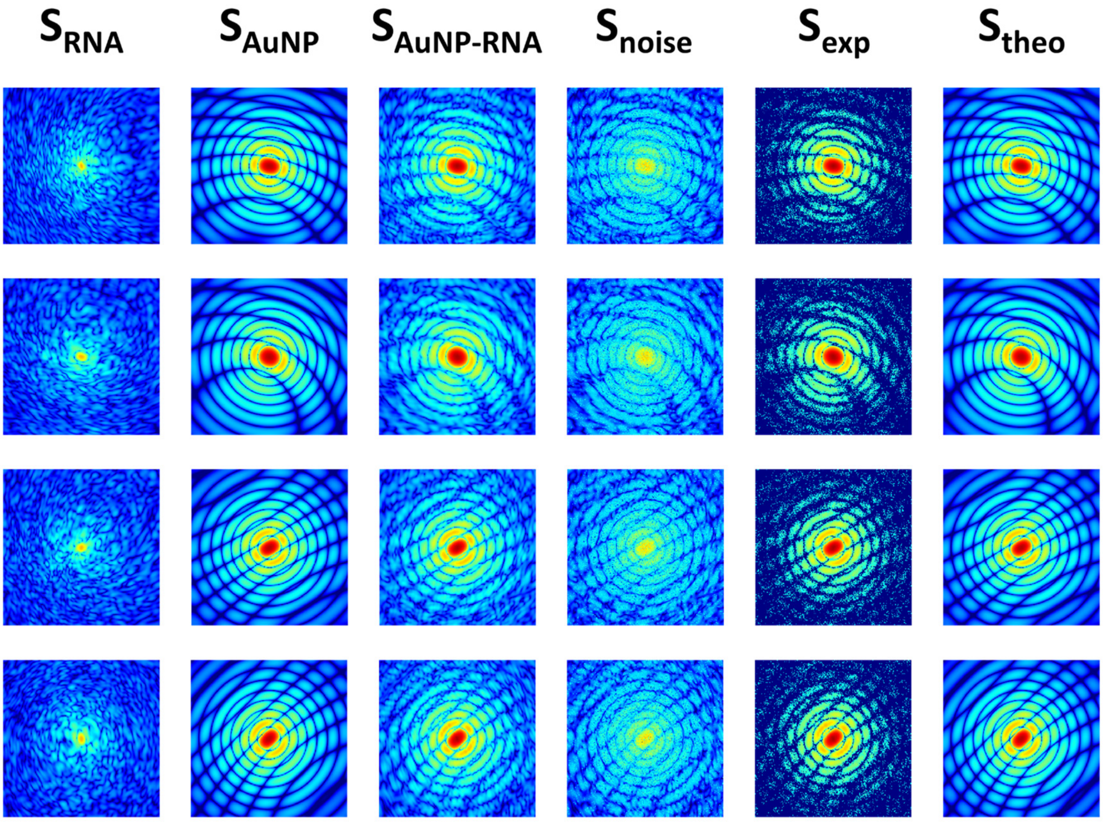

Figure S2. Various contributions to the simulated X-ray scattering images. A randomly selected pair of mock experimental (S_{exp}) and theoretical (S_{theo}) scattering images were decomposed into the contributions of each subcomponent. Four examples are presented in four rows. The first three columns visualize the scattering intensity due to a single molecule of RNA, a pair of AuNPs, and RNA labeled with two AuNPs, respectively. By adding the generated noise images (in the fourth column) to $\Delta S_{AuNP\text{–}RNA}$, we generated the mock experimental (S_{exp}) image in the fifth column. The theoretical scattering image (S_{theo}) with the optimized distance between the two AuNPs is depicted in the sixth column. S_{theo} resembles that in the second column (S_{AuNP}), but the pair distance in the former is the fitted value (r_{fit}) while that in the latter is the value from MD snapshots (r_{AuNP}). We note that the scattering intensity was logarithmically scaled to depict the weak signals in the high q as explained in **Figure S3**.

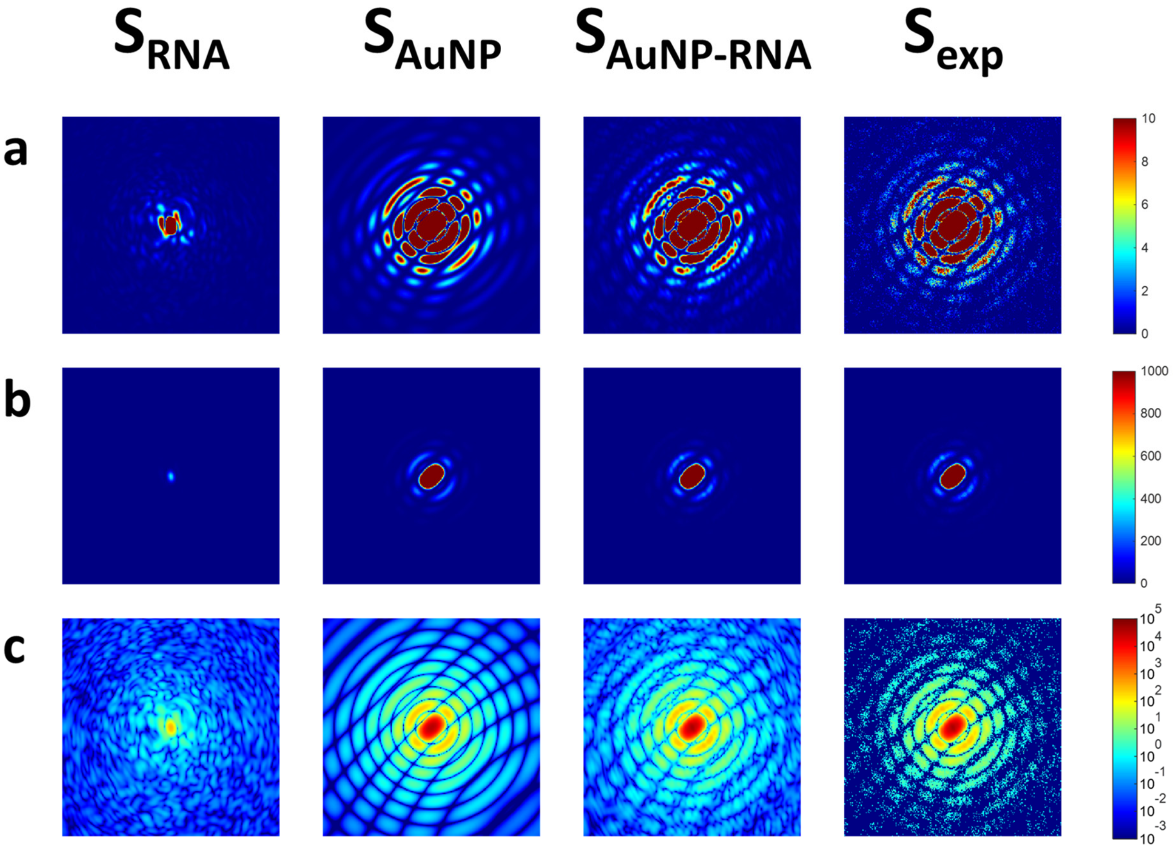

Figure S3. Visualization of X-ray scattering images in linear and logarithm of intensity. One series of X-ray scattering images in **Figure S2** are visualized with (a,b) linear and (c) logarithmic scales of intensity. The color maps used for the visualization are represented in the rightmost bar. As what can be observed from (a) and (b), in the linear intensity scale, the scattering signals are visibly interpretable at only one of the low-q (concentric circle close to the center of the detector) or the high-q (concentric circle far from the center of the detector) range. In this context, in order to concurrently visualize the scattering image across both q domains, we employed a logarithmic scale with a colormap ranging from 10^{-3} to 10^{5} in panel (c) and throughout the manuscript.

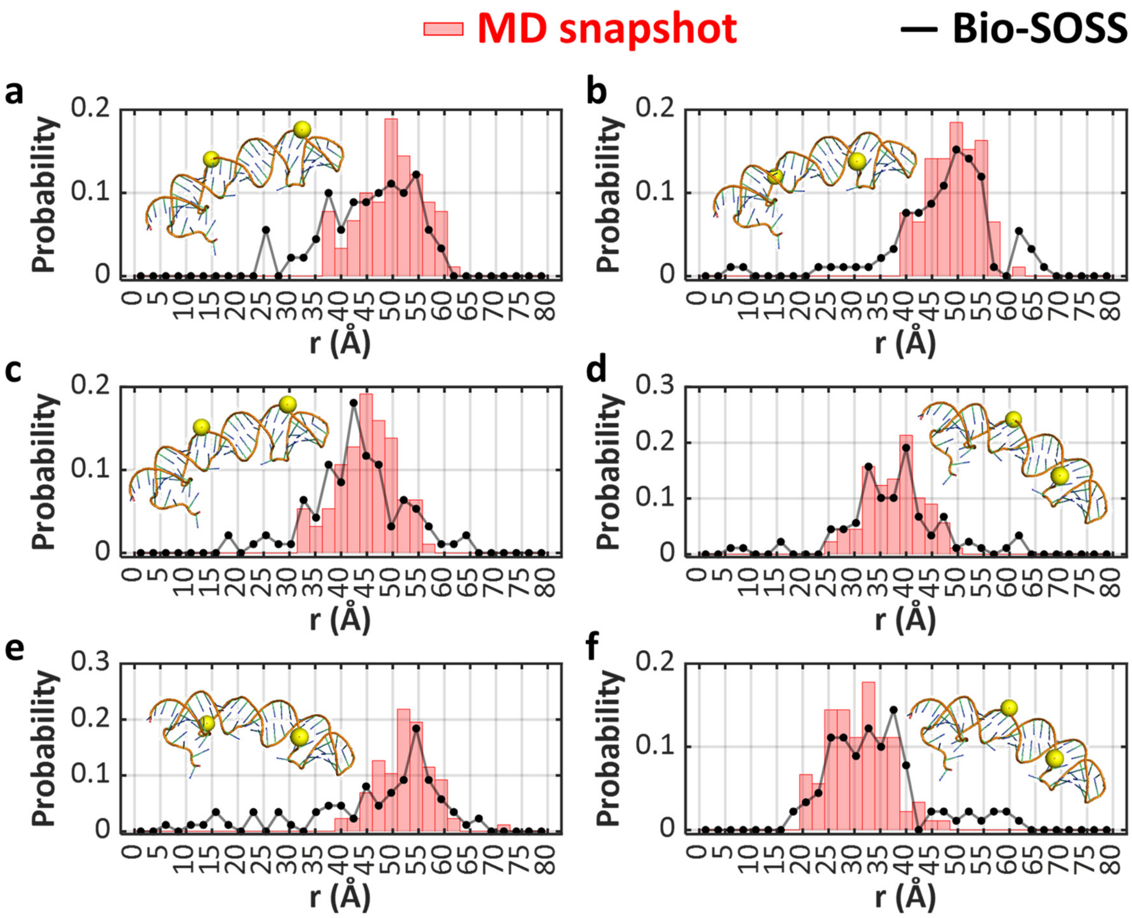

Figure S4. Evaluation of the distance distribution between selected pairs of the AuNPs retrieved by Bio-SOSS (7th to 12th) (a–f) The distribution of retrieved distances between each of the six selected pairs of AuNPs that are substituted to the oxygen atoms of the phosphate group (black dotted line) and the target distribution from MD snapshots (red histogram). The labeled positions of AuNPs in the RNA chain were visually depicted inside each histogram. We labeled the AuNP at the following residues of the target RNA structure: (a) (12nd, 15th), (b) (13rd, 15th), (c) (13rd, 16th), (d) (15th, 28th), (e) (16th, 28th), and (f) (12nd, 47th).

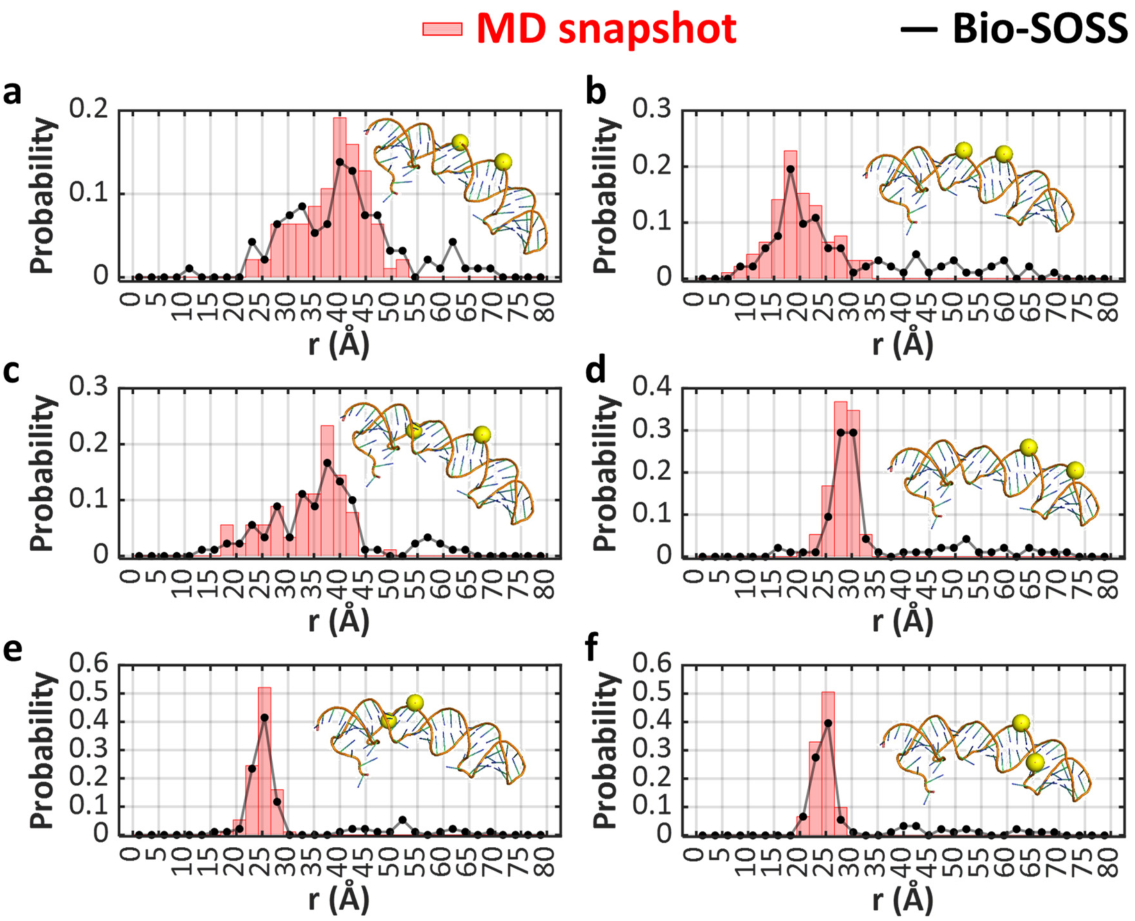

Figure S5. Evaluation of the distance distribution between selected pairs of the AuNPs retrieved by Bio-SOSS (13rd to 18th). (a–f) The distribution of retrieved distances between each of the six selected pairs of AuNPs that are substituted to the oxygen atoms of the phosphate group (black dotted line) and the target distribution from MD snapshots (red histogram). The labeled positions of AuNPs in the RNA chain were visually depicted inside each histogram. We labeled the AuNP at the following residues of the target RNA structure: (a) (13rd, 47th), (b) (16th, 47th), (c) (12nd, 50th), (d) (13rd, 50th), (e) (28th, 50th), and (f) (47th, 50th).

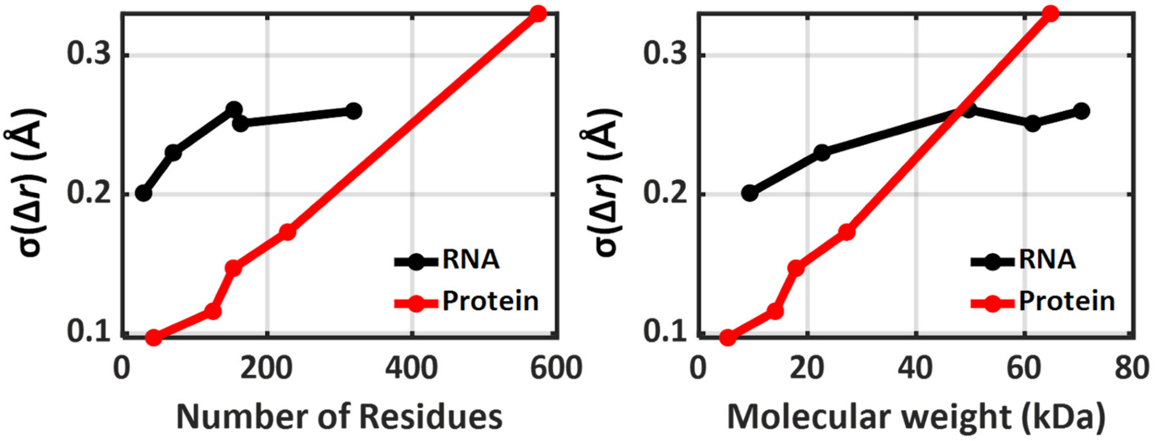

Figure S6. Dependence of the Bio-SOSS accuracy on the biomolecular size. (a) The dependence of the standard deviation of the difference distance, σ(Δr), on the number of residues. (b) The dependence of σ(Δr) on the molecular weight. In both plots, the trends of RNAs and proteins are separately portrayed in black and red solid lines, respectively. The rightmost point of the RNA plot corresponds to the RNA-protein complex (PDB ID: 1MMS). The Δr values obtained from the entire selected pairs for each biomolecule were used to calculate σ(Δr). The plots demonstrate that σ(Δr) increases proportionally with the size of the protein (or RNA), while saturation is observed in the case of large RNAs.

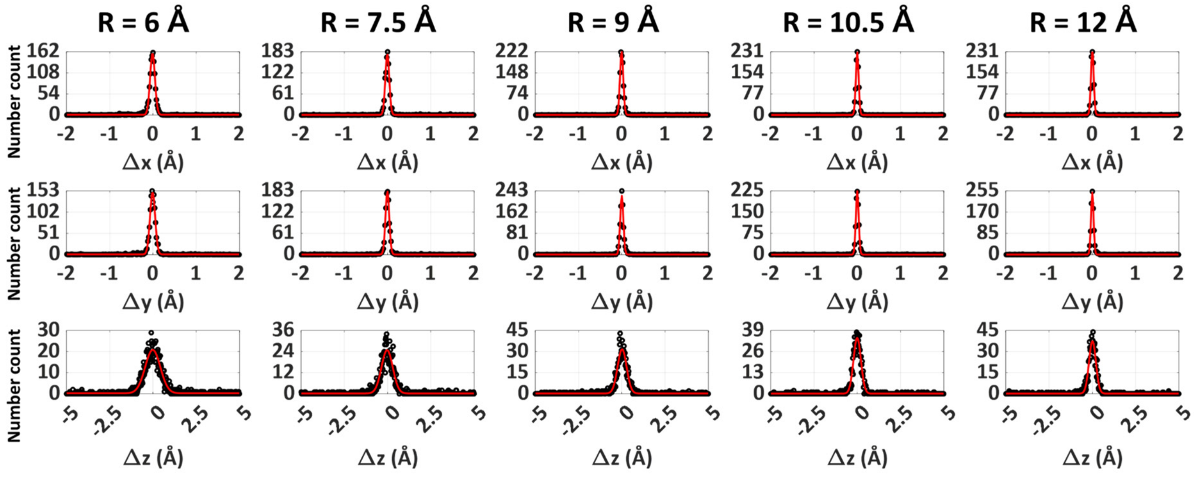

Figure S7. Gaussian fit diagrams of the distributions of difference distances at varying sizes of AuNP (larger bin). The distribution of difference distances, Δx, Δy, Δz, were sorted into a histogram of bin size Δx, Δy, Δz = 0.02 Å. The number counts at each bin were fitted by a Gaussian distribution function with mean value μ and standard deviation σ. For the Gaussian fit analysis, we used the difference distance ranges from −2 to 2 Å for Δx and Δy, and from −5 to 5 Å for Δz.

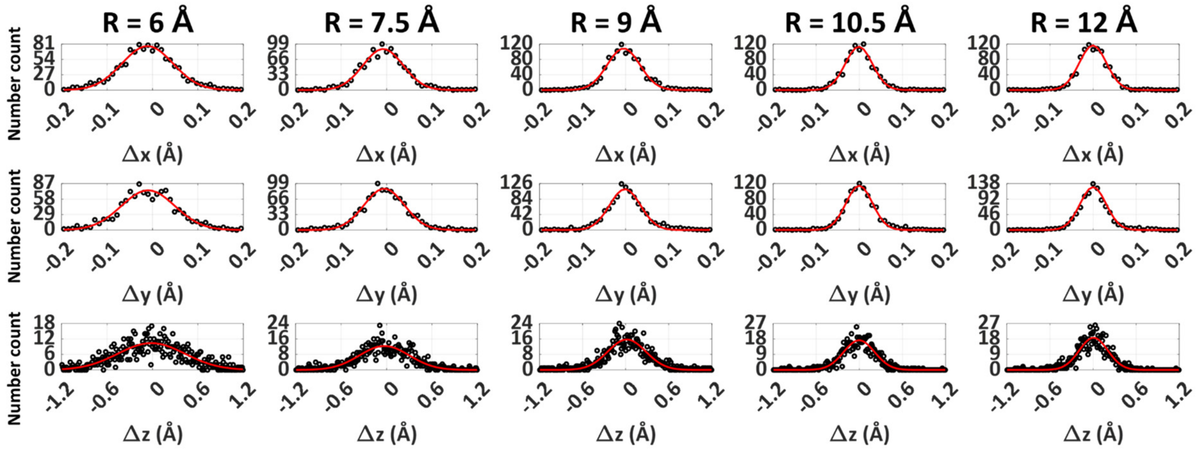

Figure S8. Gaussian fit diagrams of the distributions of difference distances at varying sizes of AuNP (smaller bin). The distribution of difference distances, Δx, Δy, Δz, were sorted into a histogram of bin size Δx, Δy, Δz = 0.01 Å. The number counts at each bin were fitted by a Gaussian distribution function with mean value μ and standard deviation σ. For the Gaussian fit analysis, we used the difference distance ranges from −0.2 to 0.2 Å for Δx and Δy, and from −1.2 to 1.2 Å for Δz.

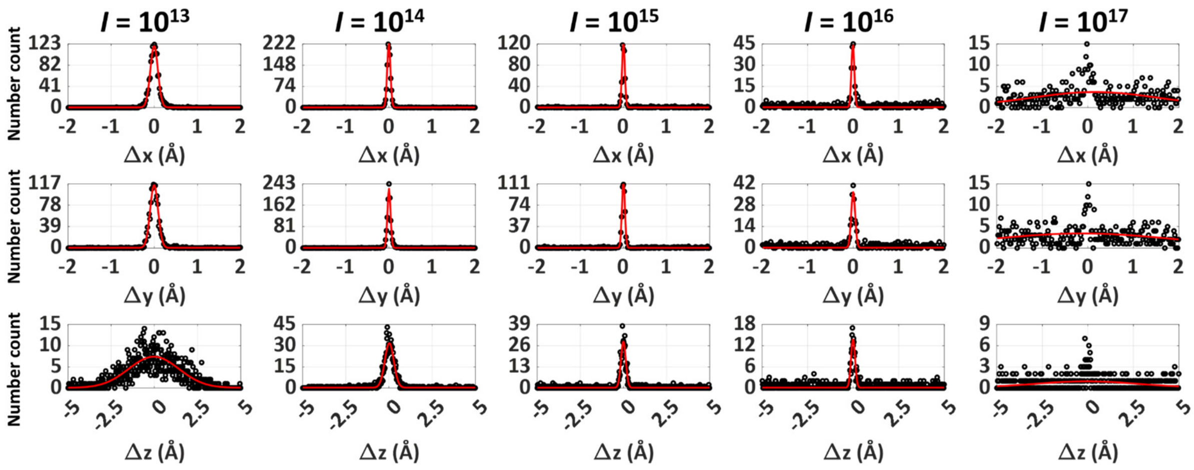

Figure S9. Gaussian fit diagrams of the distributions of difference distances at varying X-ray photon fluxes (larger bin). The distribution of difference distances, Δx, Δy, Δz, were sorted into a histogram of bin size Δx, Δy, Δz = 0.02 Å. The number counts at each bin were fitted by a Gaussian distribution function with mean value μ and standard deviation σ. For the Gaussian fit analysis, we used the difference distance ranges from −2 to 2 Å for Δx and Δy, and from −5 to 5 Å for Δz.

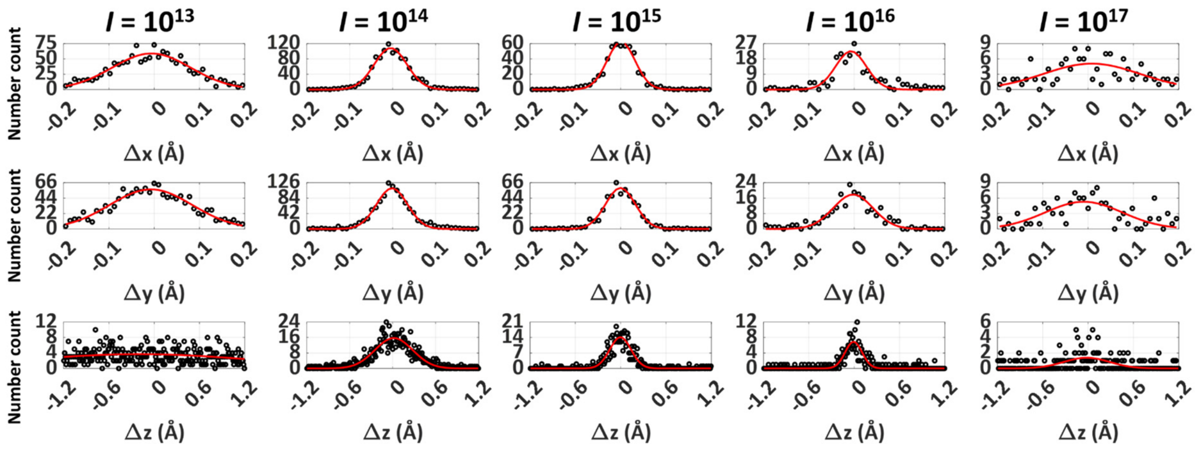

Figure S10. Gaussian fit diagrams of the distributions of difference distances at varying X-ray photon fluxes (smaller bin). The distribution of difference distances, Δx, Δy, Δz, were sorted into a histogram of bin size Δx, Δy, Δz = 0.01 Å. The number counts at each bin were fitted by a Gaussian distribution function with mean value μ and standard deviation σ. For the Gaussian fit analysis, we used the difference distance ranges from −0.2 to 0.2 Å for Δx and Δy, and from −1.2 to 1.2 Å for Δz.

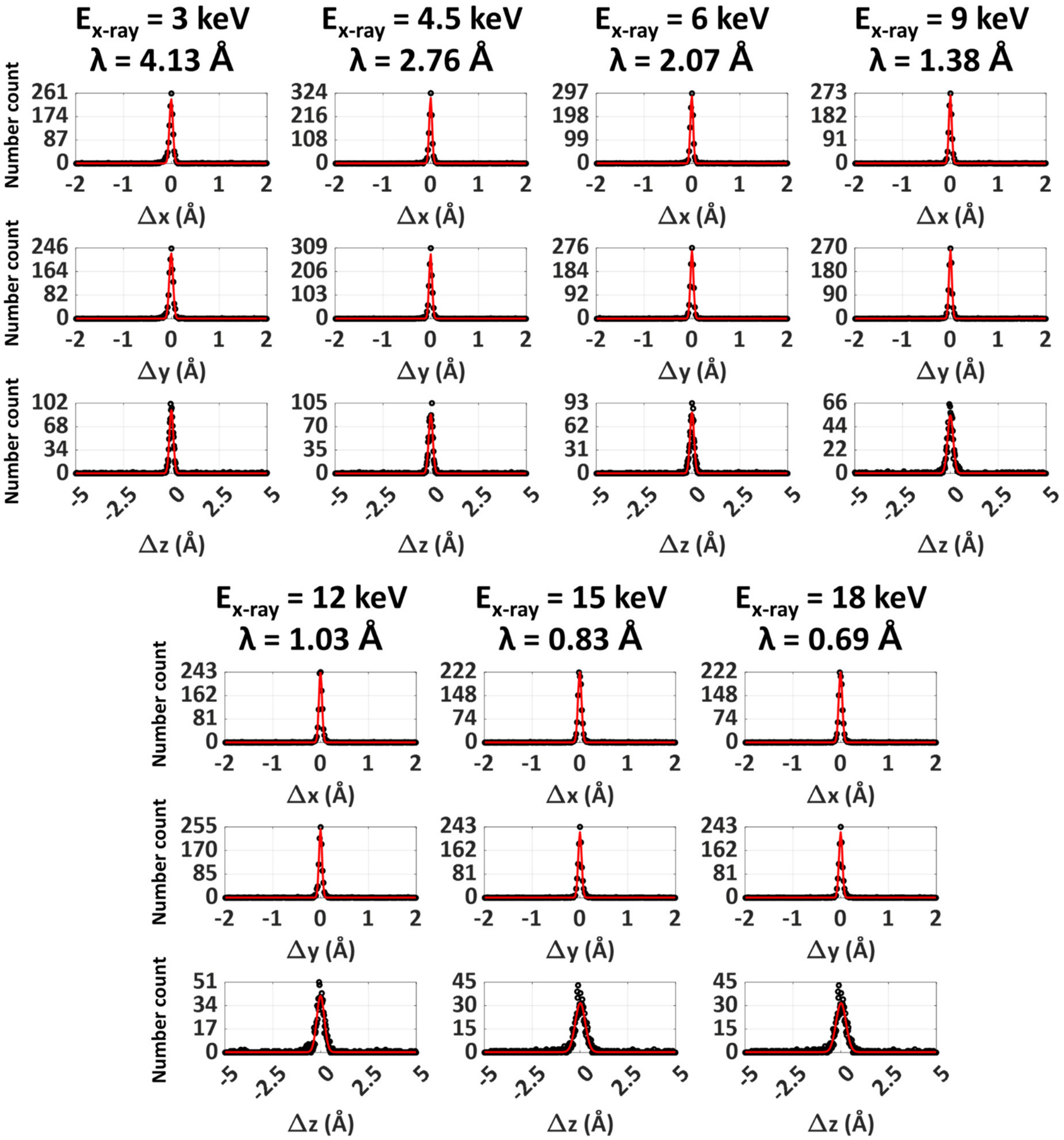

Figure S11. Gaussian fits of the distributions of difference distances at varying X-ray energies (larger bin). The distribution of difference distances, Δx, Δy, Δz, were sorted into a histogram of bin size Δx, Δy, Δz = 0.02 Å. The number counts at each bin were fitted by a Gaussian distribution function with mean value μ and standard deviation σ. For the Gaussian fit analysis, we used the difference distance ranges from −2 to 2 Å for Δx and Δy, and from −5 to 5 Å for Δz.

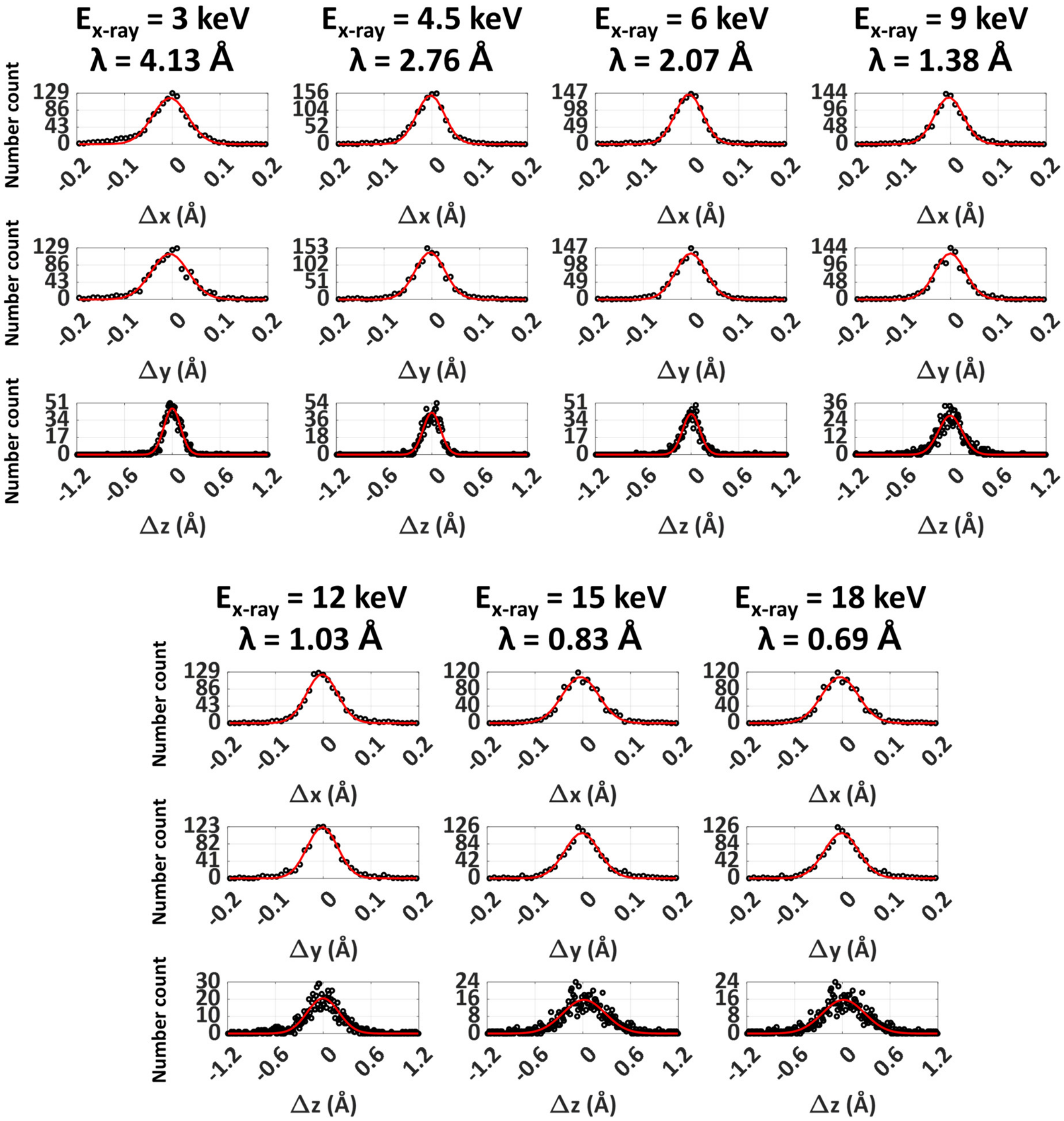

Figure S12. Gaussian fits of the distributions of difference distances at varying X-ray energies (smaller bin). The distribution of difference distances, Δx, Δy, Δz, were sorted into a histogram of bin size Δx, Δy, Δz = 0.01 Å. The number counts at each bin were fitted by a Gaussian distribution function with mean value μ and standard deviation σ. For the Gaussian fit analysis, we used the difference distance ranges from −0.2 to 0.2 Å for Δx and Δy, and from −1.2 to 1.2 Å for Δz.

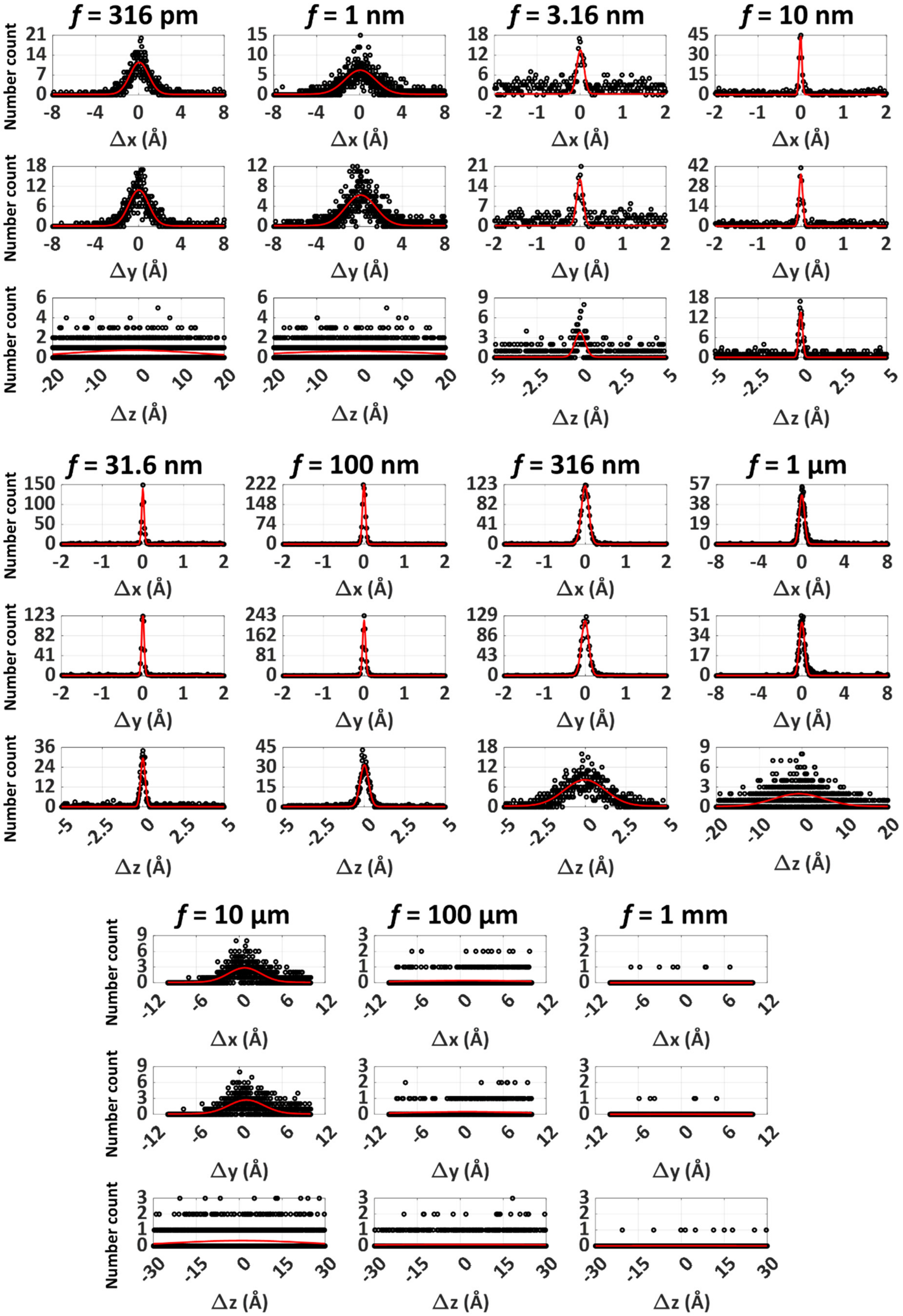

f = 316 pm
f = 1 nm
f = 3.16 nm
f = 10 nm
f = 31.6 nm
f = 100 nm
f = 316 nm
f = 1 µm
f = 10 µm
f = 100 µm
f = 1 mm
Number count
Δx (Å)
Δy (Å)
Δz (Å)

Figure S13. Gaussian fit diagrams of the distributions of difference distances at varying X-ray focal sizes. The distribution of difference distances, Δx, Δy, Δz, were sorted into a histogram of bin size Δx, Δy, Δz = 0.02 Å. The number counts at each bin were fitted by a Gaussian distribution function with mean value μ and standard deviation σ. Since the distribution varies significantly at X-ray focal sizes of interest, we used distinct ranges for the difference distance distributions at each condition. For the Gaussian fit analysis, specific difference distance ranges were employed based on the value of d: (i) Ranges from −8 to 8 Å for Δx and Δy, and from −20 to 20 Å for Δz, for d = 316 pm, 1 nm, and 1 um. (ii) Ranges from −2 to 2 Å for Δx and Δy, and from −5 to 5 Å for Δz, for d = 3.16 nm, 10 nm, 31.6 nm, 100 nm, and 316 nm, from −2 to 2 Å for Δx and Δy, and from −5 to 5 Å for Δz (d = 3.16 nm, 10 nm, 31.6 nm, 100 nm, and 316 nm. (iii) Ranges from −10 to 10 Å for Δx and Δy, and from −30 to 30 Å for Δz, for d = 10 um, 100 um, and 1 mm.

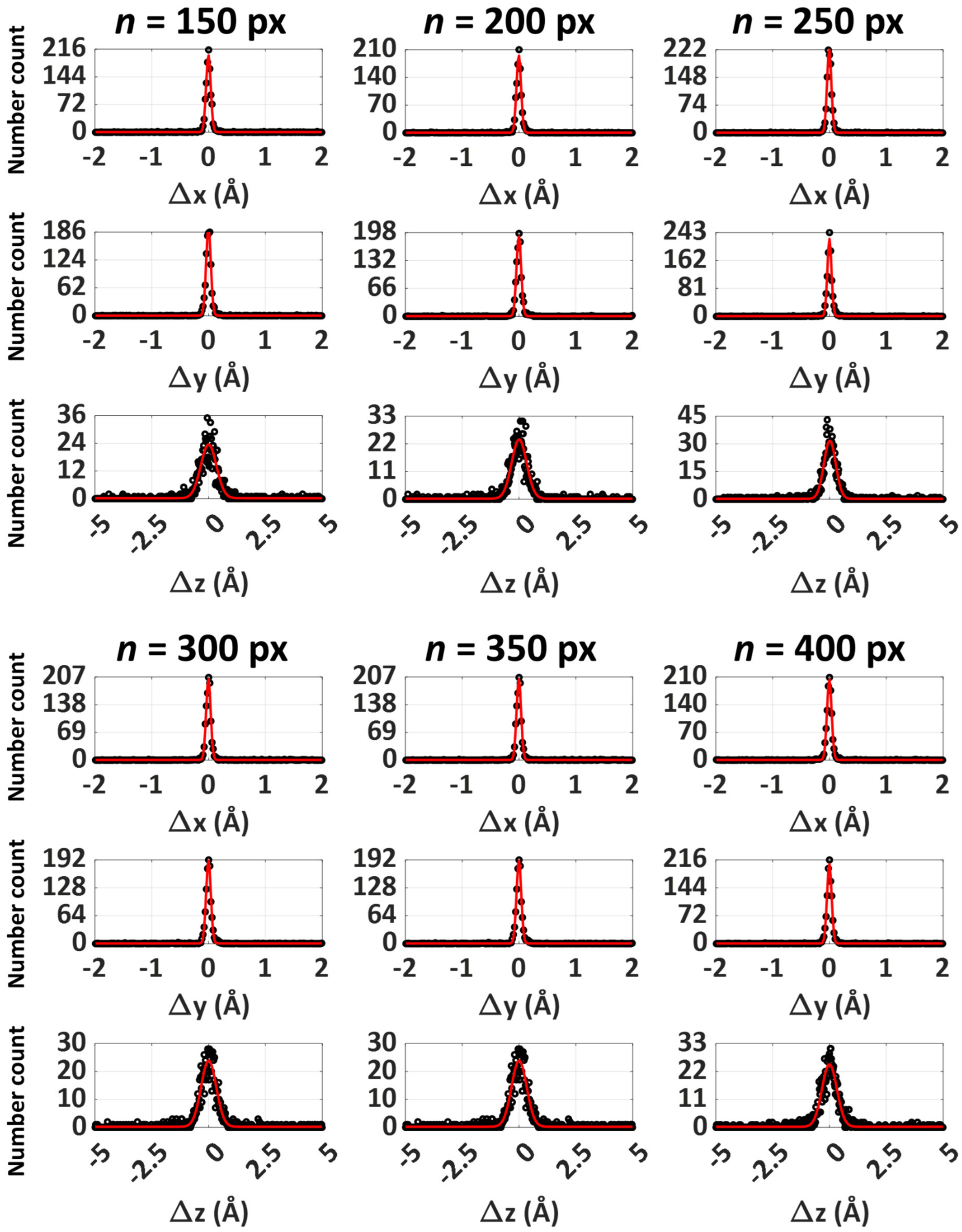

Figure S14. Gaussian fit diagrams of the distributions of difference distances at varying numbers of pixels in the detector (larger bin). The distribution of difference distances, Δx, Δy, Δz, were sorted into a histogram of bin size Δx, Δy, Δz = 0.02 Å. The number counts at each bin were fitted by a Gaussian distribution function with mean value μ and standard deviation σ. For the Gaussian fit analysis, we used the difference distance ranges from −2 to 2 Å for Δx and Δy, and from −5 to 5 Å for Δz.

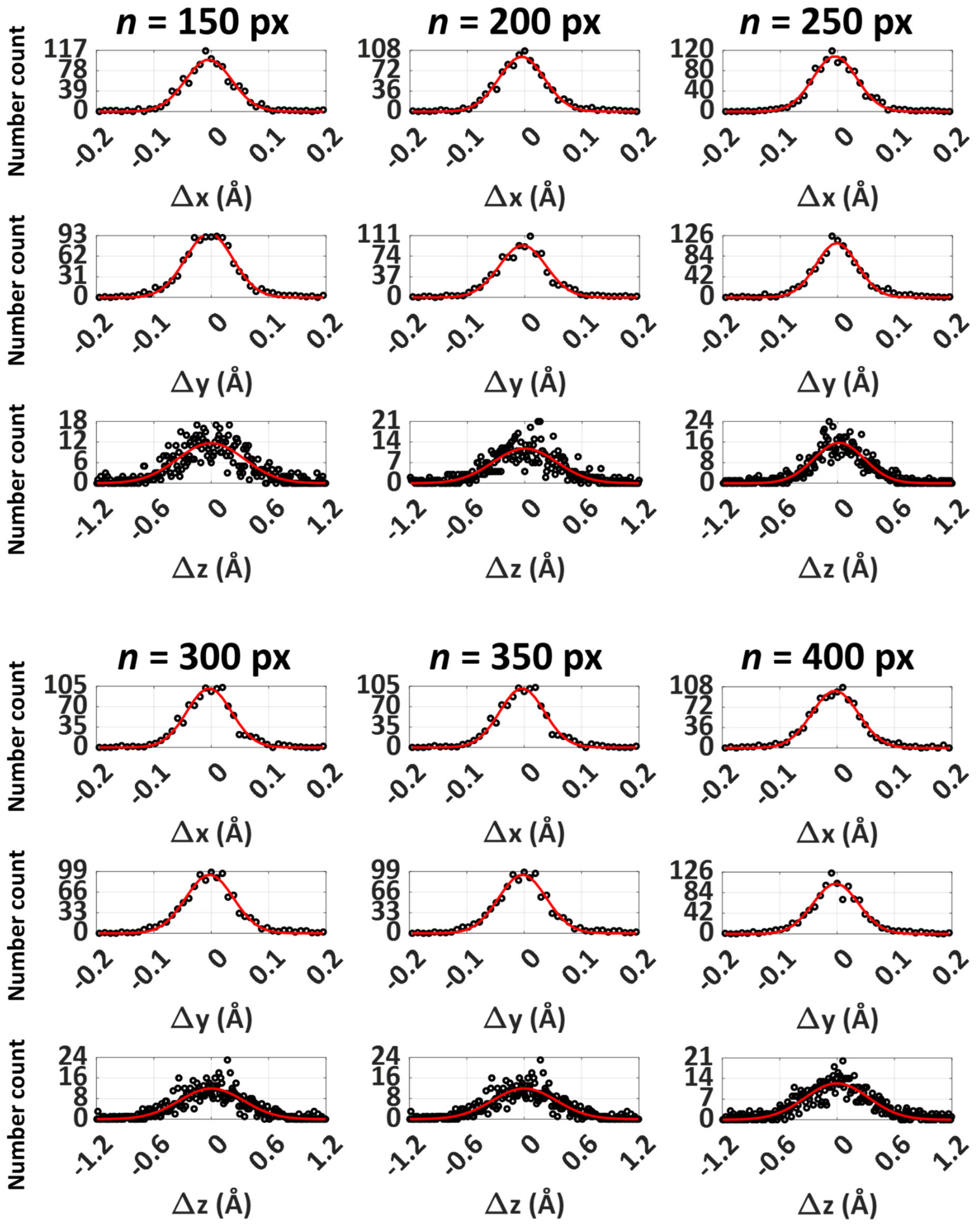

Figure S15. Gaussian fit diagrams of the distributions of difference distances at varying numbers of pixels in the detector (smaller bin). The distribution of difference distances, Δx, Δy, Δz, were sorted into a histogram of bin size Δx, Δy, Δz = 0.01 Å. The number counts at each bin were fitted by a Gaussian distribution function with mean value μ and standard deviation σ. For the Gaussian fit analysis, we used the difference distance ranges from −0.2 to 0.2 Å for Δx and Δy, and from −1.2 to 1.2 Å for Δz.

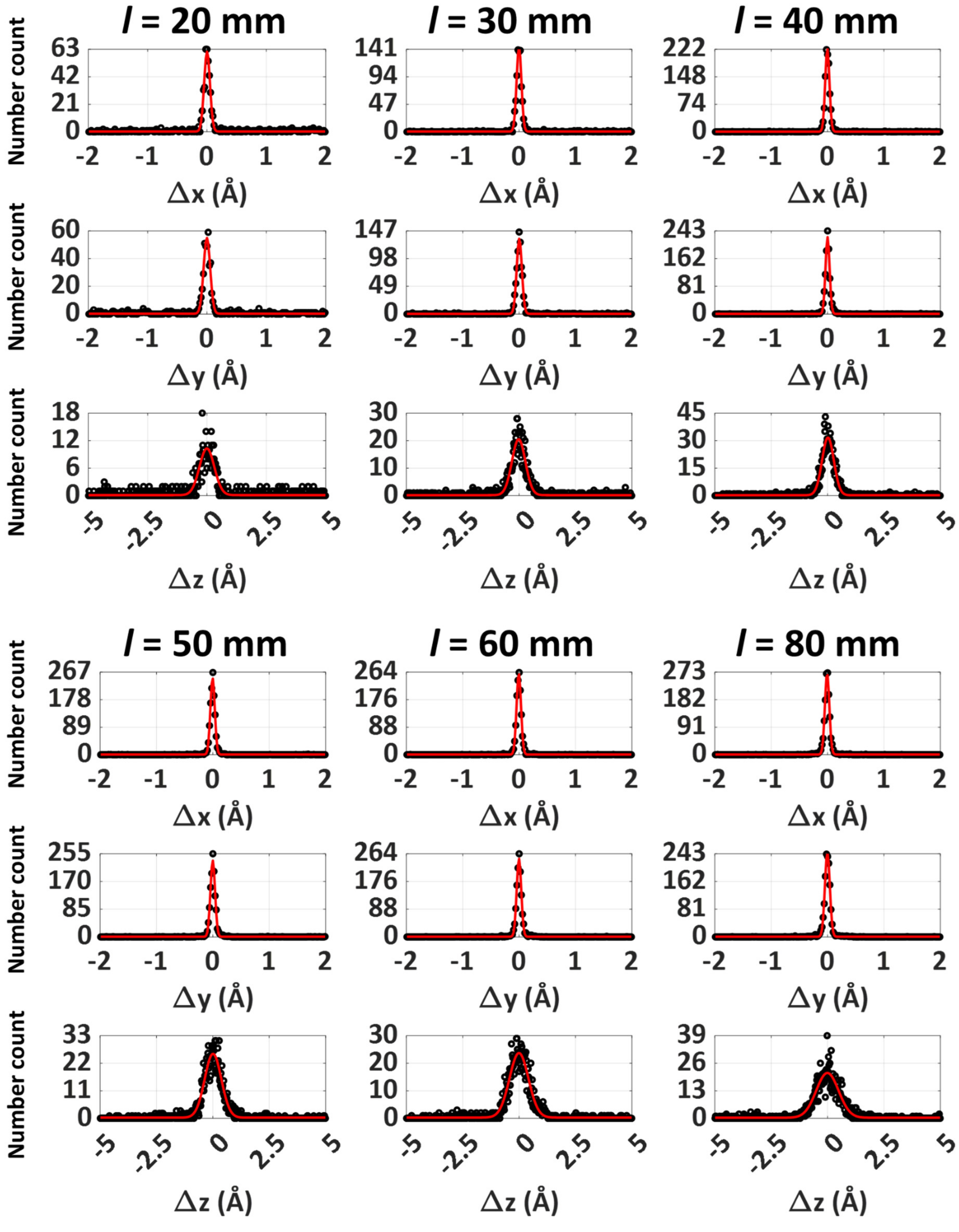

Figure S16. Gaussian fit diagrams of the distributions of difference distances at varying sample-to-detector distances (larger bin). The distribution of difference distances, Δx, Δy, Δz, were sorted into a histogram of bin size Δx, Δy, Δz = 0.02 Å. The number counts at each bin were fitted by a Gaussian distribution function with mean value μ and standard deviation σ. For the Gaussian fit analysis, we used the difference distance ranges from −2 to 2 Å for Δx and Δy, and from −5 to 5 Å for Δz.

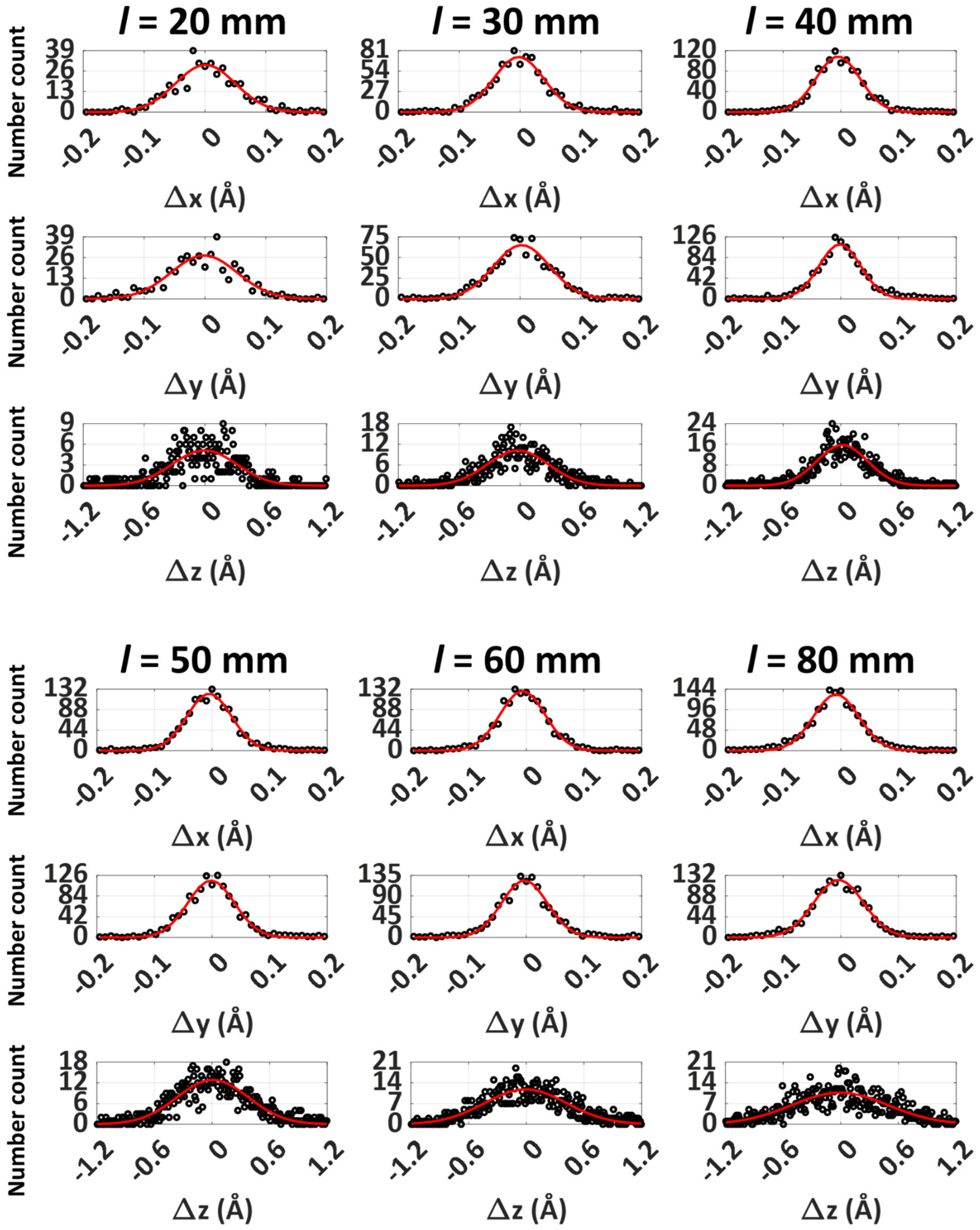

Figure S17. Gaussian fit diagrams of the distributions of difference distances at varying sample-to-detector distances (smaller bin). The distribution of difference distances, Δx, Δy, Δz, were sorted into a histogram of bin size Δx, Δy, Δz = 0.01 Å. The number counts at each bin were fitted by a Gaussian distribution function with mean value μ and standard deviation σ. For the Gaussian fit analysis, we used the difference distance ranges from −0.2 to 0.2 Å for Δx and Δy, and from −1.2 to 1.2 Å for Δz.

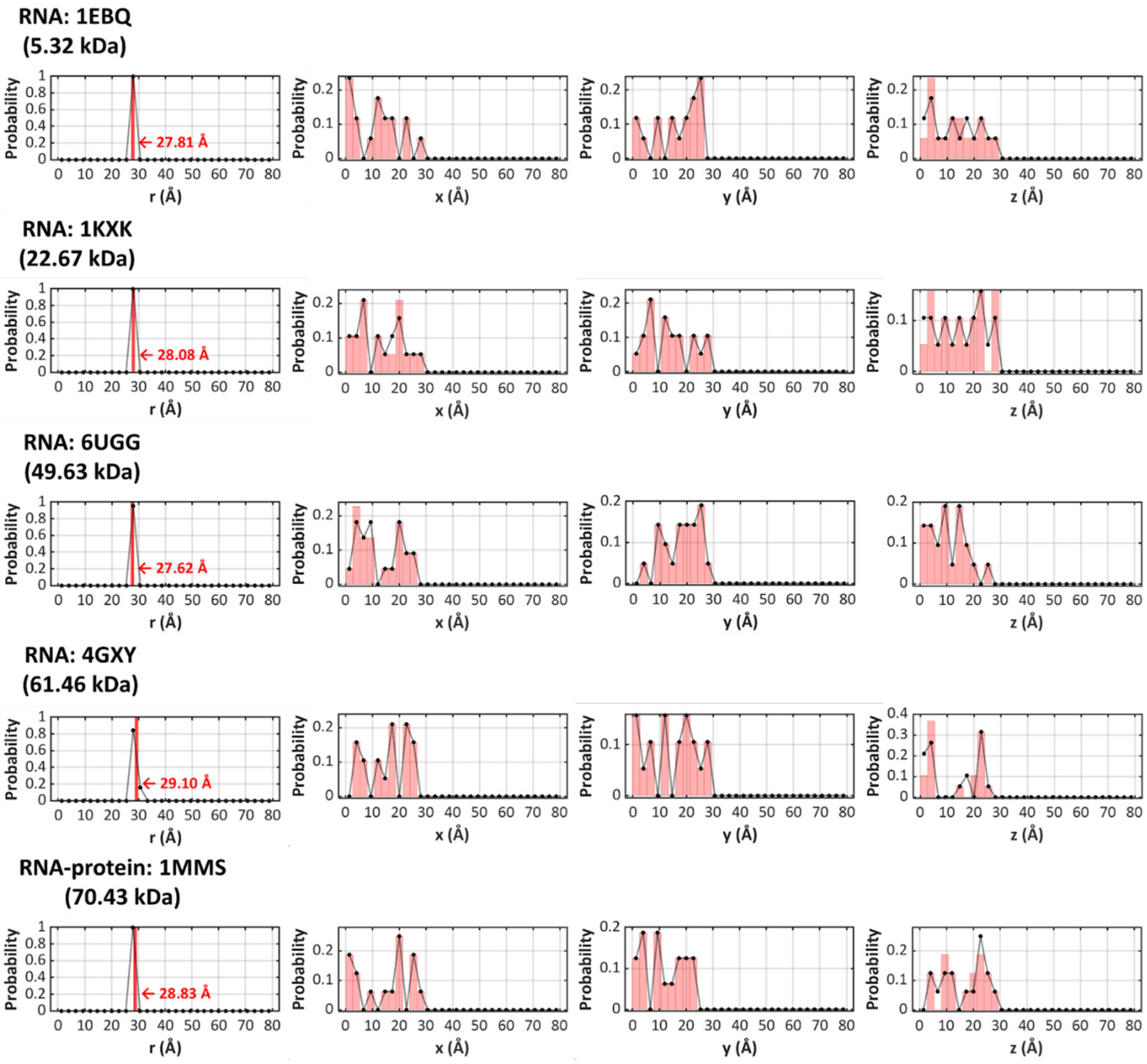

Figure S18. The distribution of distances in RNAs and an RNA-protein complex retrieved through Bio-SOSS. The distance between the two AuNP-labeled sites (r_{AuNP}, red vertical bar of the first column) are compared with the retrieved distribution of distances (r_{fit}, black line) plotted in histogram. Since we used 30 molecular orientations which share a common fixed structure, the actual distance is uniquely determined while the fitted distance can have distributions. In the second, third, and fourth columns, we plot the projection of both r_{AuNP} and r_{fit} on the three cartesian axes defined with respect to the lab frame. Among the full set of distances for each biomolecule, we chose the ones that fall within the range between 25 to 30 Å for a rigorous comparison among all RNAs and the RNA-protein complex.

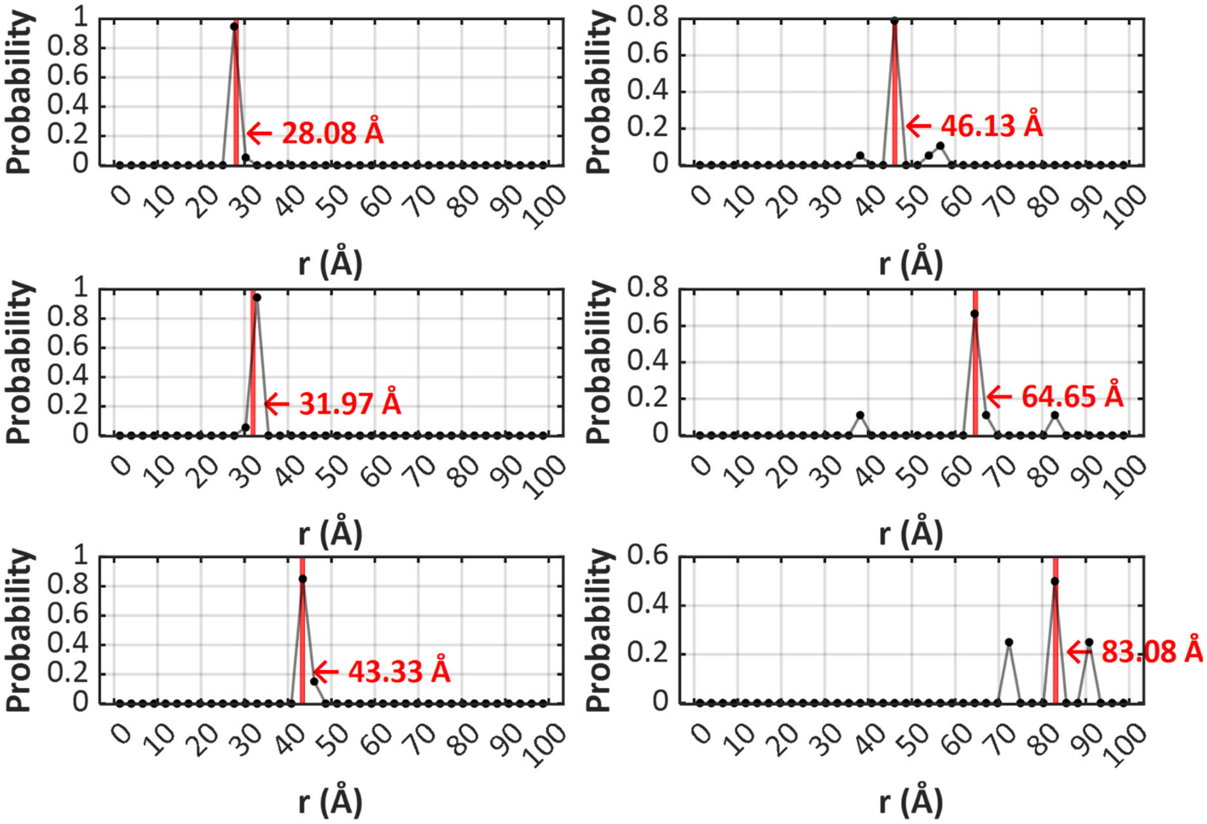

Figure S19. The distance retrieval results from six different pairs from an example RNA (PDB ID: 1KXK) through Bio-SOSS. We labeled the AuNPs at the following base indices of the target RNA structure: (29th, 40th) for 28.08 Å, (13th, 64th) for 31.97 Å, (31st, 52nd) for 43.33 Å, (16th, 42nd) for 46.13 Å, (13th, 33rd) for 64.65 Å, and (2nd, 38th) for 83.08 Å.

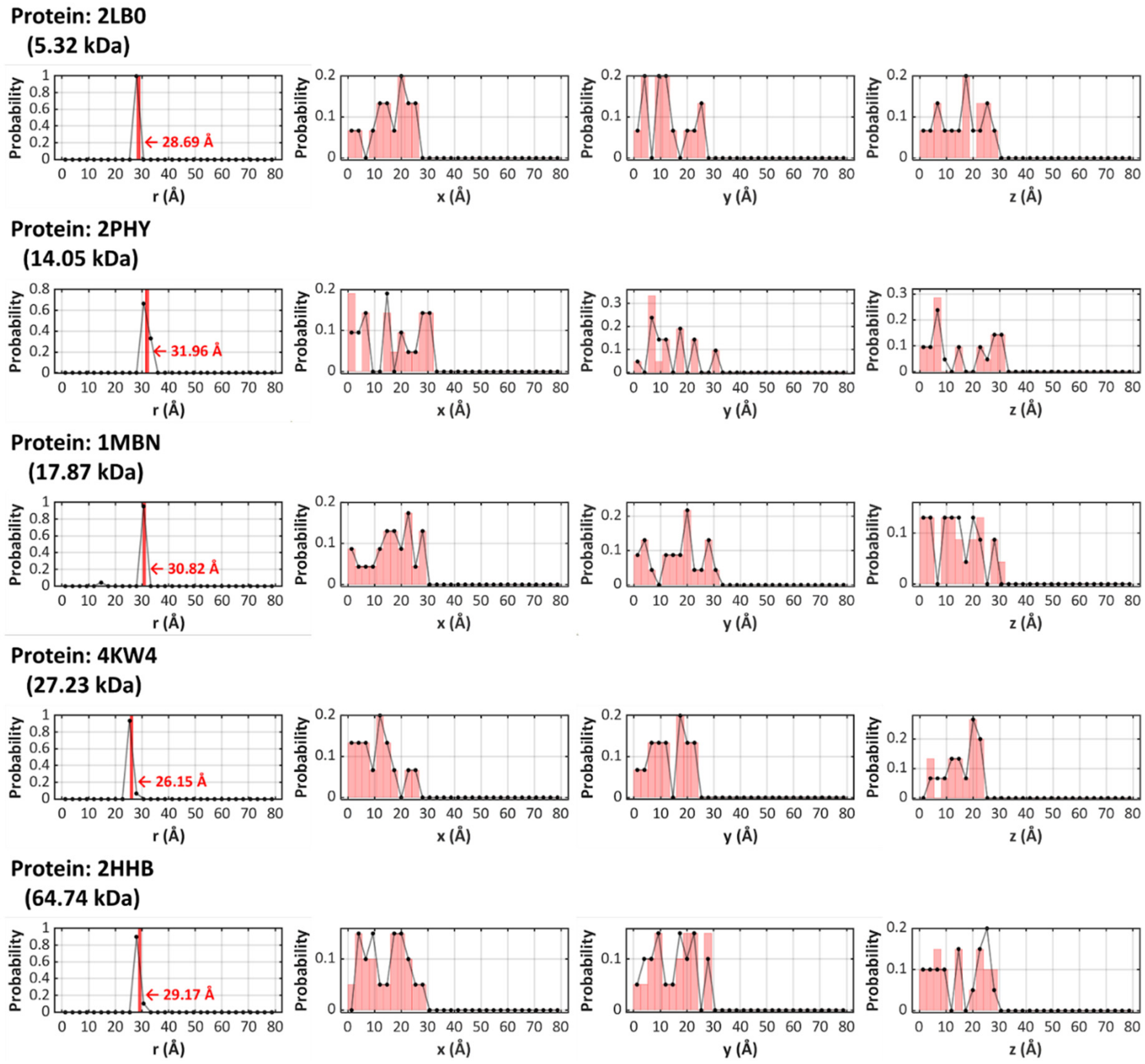

Figure S20. The distribution of distances in proteins retrieved through Bio-SOSS. The distance between the two AuNP-labeled sites (r_{AuNP}, red vertical bar of the first column) were compared with the retrieved distribution of distances (r_{fit}, black line) plotted in histogram. Since we used 30 molecular conformations which share a common fixed structure and only differ from each other in their relative orientations, the actual distance is uniquely determined while the fitted distance can have distributions. In the second, third, and fourth columns, we plot the projection of both r_{AuNP} and r_{fit} on the three cartesian axes defined with respect to the lab frame. Among the full set of distances for each biomolecule, we selected the ones that fall within the range between 25 to 30 Å for a rigorous comparison among all proteins.

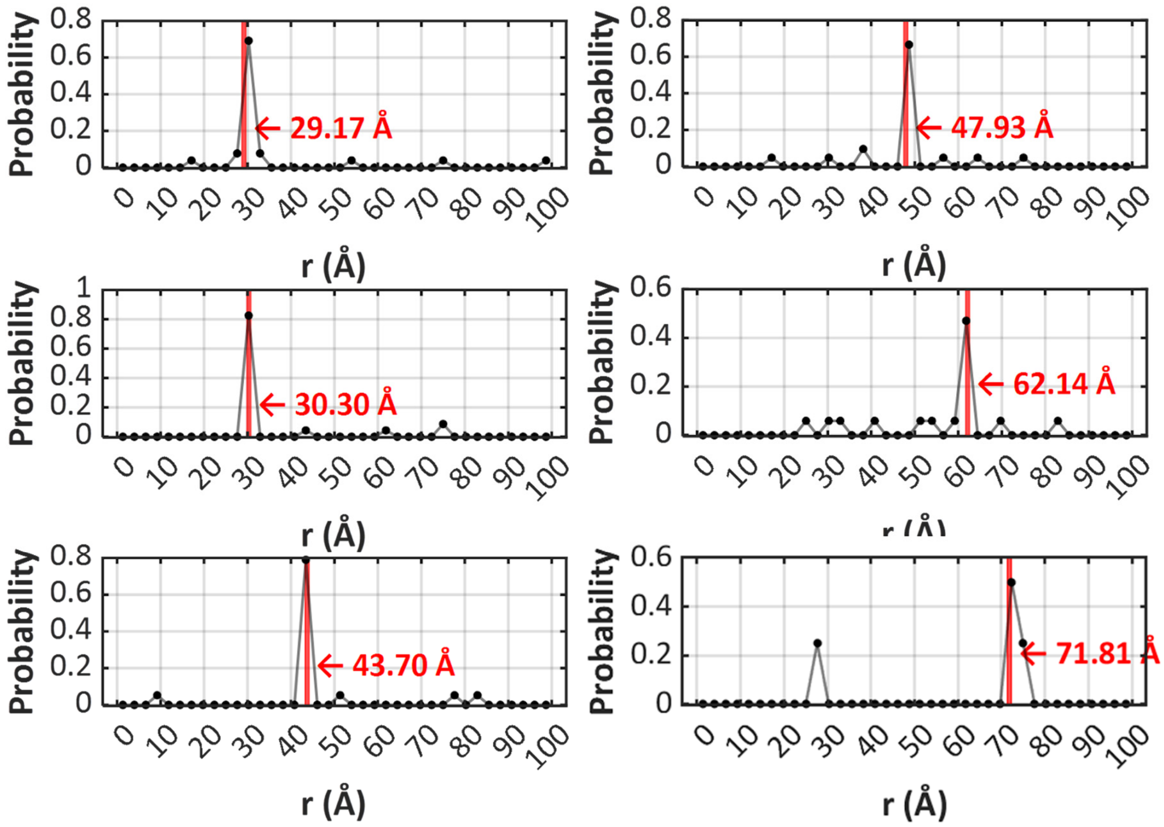

Figure S21. The distance retrieval results from six different pairs from a sample protein (PDB ID: 2HHB) through Bio-SOSS. We labeled the AuNP at the following residues of the target protein structure: (446th, 509th) for 29.17 Å, (49th, 258th) for 30.30 Å, (258st, 525th) for 43.70 Å, (43rd, 446th) for 47.93 Å, (157th, 479th) for 62.14 Å, and (157th, 547th) for 71.81 Å.

In this section, Supplementary Tables

Table S1. Quantitative results of Bio-SOSS conducted for four RNAs (PDB IDs: 1EBQ, 1XKX, 6UGG, 4GXY) and one RNA-protein complex (PDB ID: 1MMS). The simulation was conducted on 30 differently oriented structures of a single static PDB structure for each biomolecule. The standard deviation of the distance differences, σ(Δr), were extracted and plotted in **Figure S6**.

	RNA				RNA-protein
	1EBQ	1KXK	6UGG	4GXY	1MMS
Number of atoms	619	1499	3311	3685	4173
Number of nucleotides	29	70	154	163	319
Molecular weight (kDa)	9.36	22.67	49.63	61.46	70.43
Minimum r_{AuNP} (Å)	27.8	28.1	27.6	24.6	21.5
Maximum r_{AuNP} (Å)	50.1	83.1	92.0	91.1	88.3
Number of tested pairs	4	6	6	6	6
σ(Δr) (Å)	0.20 ± 0.021	0.20 ± 0.019	0.26 ± 0.026	0.25 ± 0.032	0.26 ± 0.036

Table S2. Quantitative results of Bio-SOSS conducted for five proteins (PDB IDs: 2LB0, 2PHY, 1MBN, 4KW4, 2HHB). The simulation was conducted on the 30 differently oriented structures of a single static PDB structure for each biomolecule. The standard deviation of the distance differences, σ(Δr), were extracted and plotted in **Figure S6**.

	Protein				
	2LB0	2PHY	1MBN	4KW4	2HHB
Number of atoms	355	1114	1260	2175	4779
Number of nucleotides	43	125	153	228	574
Molecular weight (kDa)	5.32	14.05	17.87	27.23	64.74
Minimum r_{AuNP} (Å)	22.9	25.5	30.8	18.4	29.2
Maximum r_{AuNP} (Å)	28.7	42.3	48.1	49.9	71.8
Number of tested pairs	2	4	4	4	6
σ(Δr) (Å)	0.097 ± 0.017	0.12 ± 0.022	0.15 ± 0.012	0.17 ± 0.016	0.33 ± 0.038

The citation indices in **Figure S1** are replicated from the main manuscript.

47. Peterson, R.D.; Feigon, J. Structural change in Rev responsive element RNA of HIV-1 on binding Rev peptide. *J. Mol. Biol.* **1996**, 264, 863-877, https://doi.org/10.1006/jmbi.1996.0683.
48. Zhang, L.; Doudna, J.A. Structural insights into group ii intron catalysis and branch-site selection. *Science* **2002**, 295, 2084-2088, https://doi.org/10.1126/science.1069268.
49. Chan, C.W.; Badong, D.; Rajan, R.; Mondragón, A. Crystal structures of an unmodified bacterial tRNA reveal intrinsic structural flexibility and plasticity as general properties of unbound tRNAs. *RNA* **2020**, 26, 278-289, https://doi.org/10.1261/rna.073478.119.
50. Peselis, A.; Serganov, A. Structural insights into ligand binding and gene expression control by an adenosylcobalamin riboswitch. *Nat. Struct. Mol. Biol.* **2012**, 19, 1182-1184, https://doi.org/10.1038/nsmb.2405.
51. Wimberly, B.T.; Guymon, R.; McCutcheon, J.P.; White, S.W.; Ramakrishnan, V. A detailed view of a ribosomal active site: the structure of the L11–RNA complex. *Cell* **1999**, 97, 491-502, https://doi.org/10.1016/S0092-8674(00)80759-X.
52. Aragón, E.; Goerner, N.; Zaromytidou, A.-I.; Xi, Q.; Escobedo, A.; Massagué, J.; Macias, M.J. A Smad action turnover switch operated by WW domain readers of a phosphoserine code. *Genes & Development* **2011**, 25, 1275-1288, https://doi.org/10.1101/gad.2060811.
53. Borgstahl, G.E.O.; Williams, D.R.; Getzoff, E.D. 1.4 Å structure of photoactive yellow protein, a cytosolic photoreceptor: Unusual fold, active site, and chromophore. *Biochemistry* **1995**, 34, 6278-6287, https://doi.org/10.1021/bi00019a004.
54. Watson, H.C.; Kendrew, J. The stereochemistry of the protein myoglobin. *Prog. Stereochem.* **1969**, *4*, 299. https://doi.org/10.2210/pdb1mbn/
55. Yang, F.; Moss, L.G.; Phillips, G.N., Jr. The molecular structure of green fluorescent protein. *Nat. Biotechnol.* **1996**, 14, 1246-1251, https://doi.org/10.1038/nbt1096-1246.
56. Fermi, G.; Perutz, M.F.; Shaanan, B.; Fourme, R. The crystal structure of human deoxyhaemoglobin at 1.74 Å resolution. *J. Mol. Biol.* **1984**, 175, 159-174, https://doi.org/10.1016/0022-2836(84)90472-8.
